# The Art of Positronics in Contemporary Nanomaterials Science: A Case Study of Sub-Nanometer Scaled Glassy Arsenoselenides

**DOI:** 10.3390/ma15010302

**Published:** 2022-01-01

**Authors:** Oleh Shpotyuk, Adam Ingram, Catherine Boussard-Pledel, Bruno Bureau, Zdenka Lukáčová Bujňáková, Peter Baláž, Bohdan Mahlovanyi, Yaroslav Shpotyuk

**Affiliations:** 1 Department of Optical Glass and Ceramics, Vlokh Institute of Physical Optics, 23 Dragomanov Str., 79005 Lviv, Ukraine; olehshpotyuk@yahoo.com; 2 Faculty of Science and Technology, Jan Dlugosz University in Czestochowa, 13/15 al. Armii Krajowej, 42-200 Czestochowa, Poland; 3 Department of Physics, Opole University of Technology, 75 Ozimska Str., 45-370 Opole, Poland; a.ingram@po.opole.pl; 4 CNRS, ISCR, Université de Rennes, UMR 6226, F-35000 Rennes, France; catherine.boussard@univ-rennes1.fr (C.B.-P.); bruno.bureau@univ-rennes1.fr (B.B.); bmahlovanyi@ur.edu.pl (B.M.); 5 Institute of Geotechnics of Slovak Academy of Sciences, 45 Watsonova Str., 04001 Košice, Slovakia; bujnakova@saske.sk (Z.L.B.); balaz@saske.sk (P.B.); 6 Institute of Physics, University of Rzeszow, 1 Pigonia Str., 35-959 Rzeszow, Poland; 7 Department of Sensor and Semiconductor Electronics, Ivan Franko National, University of Lviv, 107 Tarnavskoho Str., 79017 Lviv, Ukraine

**Keywords:** nanocomposites, mechanomilling, volumetric nanostructurization, glass, positron annihilation lifetime spectroscopy

## Abstract

The possibilities surrounding positronics, a versatile noninvasive tool employing annihilating positrons to probe atomic-deficient sub-nanometric imperfections in a condensed matter, are analyzed in application to glassy arsenoselenides g-As_x_Se_100−x_ (0 < x < 65), subjected to dry and wet (in 0.5% PVP water solution) nanomilling. A preliminary analysis was performed within a modified two-state simple trapping model (STM), assuming slight contributions from bound positron–electron (Ps, positronium) states. Positron trapping in g-As_x_Se_100−x_/PVP nanocomposites was modified by an enriched population of Ps-decay sites in PVP. This was proven within a three-state STM, assuming two additive inputs in an overall trapping arising from distinct positron and Ps-related states. Formalism of x3-x2-CDA (coupling decomposition algorithm), describing the conversion of Ps-decay sites into positron traps, was applied to identify volumetric nanostructurization in wet-milled g-As-Se, with respect to dry-milled ones. Under wet nanomilling, the Ps-decay sites stabilized in inter-particle triple junctions filled with PVP replaced positron traps in dry-milled substances, the latter corresponding to multi-atomic vacancies in mostly negative environments of Se atoms. With increased Se content, these traps were agglomerated due to an abundant amount of Se-Se bonds. Three-component lifetime spectra with nanostructurally- and compositionally-tuned Ps-decay inputs and average lifetimes serve as a basis to correctly understand the specific “rainbow” effects observed in the row from pelletized PVP to wet-milled, dry-milled, and unmilled samples.

## 1. Introduction

Chalcogenide compounds represented by glassy arsenoselenides, g-As_x_Se_100−x_, from “pure” selenium g-Se to stoichiometric g-As_40_Se_60_ (the group of under-stoichiometric arsenoselenides) and stretched further with As content beyond arsenic triselenide g-As_2_Se_3_ (viz. g-As_40_Se_60_), to As-rich g-As_65_Se_35_ (the group of over-stoichiometric arsenoselenides), compose an important family of archetypal media possessing widespread application in optoelectronics, IR photonics, opto- and space telecommunication, bio- and chemical sensing, etc. [1,2,3]. Nowadays, these disordered materials have also attracted interest in biomedicine because of their promising usage as anti-cancer drugs in the treatment of many hematological malignant diseases [4,5].

To a great extent, the medicinal usage of these compounds in contemporary therapeutics (often named *arsenicals* [4]) is tuned externally through nanostructurization, i.e., the technology reconstructing these materials over atomic and sub-atomic scales by influence of external stimuli, such as mechanochemistry [6,7,8,9], the high-energy mechanical milling (referred to as nanomilling, NM), ensuring unique biocompatibility in the material under treatment due to the abnormally enhanced ratio of surface-to-bulk states [10,11,12]. The NM is concomitant with generation of a vast variety of free-volume elements (FVE) in nanostructured material, such as vacancies, vacancy clusters and agglomerates, dislocations, some interfacial (IF) imperfections, such as inter-particle triple junctions (TJ), grain-boundaries (GB), etc., which possess an excess of atomic-deficient space as compared with non-defective close-packed conformations. In anti-microbial applications, the milled arsenicals are often combined with organic stabilizers, such as polyvinylpyrrolidone (PVP), keeping individuality of the shaped nanoparticles (NP), thus forming the PVP-capped nanocomposites [8,9,13,14,15].

In this work, atomic-deficient structures of glassy arsenoselenides, g-As_x_Se_100−x_, from glass-forming regions, covering under-stoichiometric (0 < x < 40) and over-stoichiometric (40 < x < 65) domains activated by NM in dry and wet mode (i.e., milled in PVP medium), was comprehensively analyzed, employing *positronics*, recognized as an advanced high-informative noninvasive tool probing sub-nm-scaled volumetric effects in a condensed matter, grounded on positron (e^+^) annihilation lifetime (PAL) spectroscopy [16,17,18,19,20,21,22,23,24,25,26].

## 2. Materials and Methods

### 2.1. Nanocomposite Preparation and Microstructure Characterization

The nanocomposites were prepared using coarse-grained powdered (cgp) As_x_Se_100−x_ synthesized by melt-quenching from high-purity commercial elemental precursors (As and Se of 5N purity), as described in more detail elsewhere [1,2]. The cgp samples were pelletized via a conventional procedure of compressing in stainless-steel die (under ~0.7 GPa) to produce discs (~6 mm in diameter and ~1 mm in thickness), hereafter referred to as unmilled samples.

The prepared cgp g-As_x_Se_100−x_ samples were nanostructurized by NM in dry and wet modes. The Pulverissete 6 (Fritsch, Idar-Oberstein, Germany) planetary ball mill operated at 500 rpm and protective Ar atmosphere was used for dry-NM. This procedure was performed for 20 min in a 250 mL tungsten carbide chamber (loaded with 50 tungsten carbide balls, each 10 mm in diameter), using 3 g of cgp g-As_x_Se_100−x_ sieved under 200 μm. The prepared fine-grained powdered (fgp) arsenoselenides were pelletized via the same procedure as in the case of the unmilled probes.

The part of preliminary dry-milled arsenoselenides was additionally milled in a wet mode. This 90-min attrition route was performed in the presence of 300 mL of 0.5% PVP solution using laboratory MiniCer mill (Netzsch, Selb, Germany) operated at 3500 rpm (85% loading of milling shaft with yttrium-stabilized ZrO_2_ balls, each having 0.6 mm in diameter). The powdered PVP with an average molecular weight of M_w_ = 40,000 g·mol^−1^ purchased from Sigma-Aldrich Co. LLC (St. Louis, MO, USA) was used. The nanosuspension was dried at 70 °C, and further pelletized under the same conditions, thus producing fgp wet-milled glassy arsenoselenides, representing themselves as g-As_x_Se_100−x_/PVP nanocomposites.

The NP-size distribution in the nanosuspensions identified with photon cross-correlation spectroscopy using a Nanophox (Sympatec, Germany) analyzer shows single-mode dependence with d_50_ = ~150–200 nm (i.e., 50% of NP were smaller than 150–200 nm) and d_99_ = ~250–280 nm (i.e., 99% of NP were smaller than 250–280 nm). As it follows from an X-ray diffraction analysis [8,9], the high-energy mechanical milling under the above parameters does not change the principally amorphous state of arsenicals, while essentially enhancing their degree of amorphization.

### 2.2. The PAL Spectra Recording and Preliminary Treatment

The PAL spectra were recorded with a conventional fast–fast coincidence system (ORTEC), 230 ps in resolution, which was stabilized at a temperature of 22 °C and relative humidity of 35% (for more details of the PAL spectra measurements, see reference [19]). To ensure precise positron lifetimes, each spectrum was recorded in normal measuring statistics involving as high as ~10^6^ coincidences, the channel width of 6.15 ps being chosen to allow 8000 channels in a total per spectrum. The ^22^Na isotope of low ~50-kBq activity prepared from aqueous ^22^NaCl solution (wrapped by 12 μm Kapton^®^ foil and sealed) was used as the positron source sandwiched between two samples. The source correction (12% contribution with a 0.372 ns lifetime, installed due to calibration with Ni and Kapton^®^ foil) was applied to compensate the input, originating from annihilation in the source itself and Kapton^®^ foil.

The best fitting of the collected PAL spectra was achieved with the LT 9.0 program [25] under decomposition into three constraint-free negative exponentials (so-called unconstrained x3-term fitting) obeying normalization by component intensities (*I*_1_ + *I*_2_ + *I*_3_ = 1) and stabilizing the model-independent average positron lifetime *τ_av_*^Σ^ as the mass center of the PAL spectrum:

(1) $$\tau_{av}^{\Sigma }=\overset{}{\underset{i}{\sum }}I_{i}\tau_{i}$$

where *τ_i_* and *I_i_* denote positron lifetime and intensity of the respective fitting component (*i* = 3).

This approach covers different channels in substances arising from positrons annihilating in defect-free bulk (DFB) states, intrinsic trapping sites (captured in free-volume defects), and bound positron–electron (e^+^e^−^) states (i.e., captured in positronium Ps-decay holes) [18,19,20,24]. For highly inhomogeneous nanosubstances, the unconstrained x3-term fitting to the raw PAL spectra has an obvious advantage, i.e., not resulting in any artifacts related to fixing in *τ_i_* lifetimes and *I_i_* intensities [27,28,29]. Under this preliminary step, the reconstructed PAL spectra allow the error-bar in lifetimes *τ_i_* and intensities *I_i_* at the level of ±0.005 ns and 0.5%, respectively.

### 2.3. Algorithmization of the Multi-Channel PAL Spectra in Nanocomposites

In nanostructurized heterogeneous substances, such as *multi-nanoparticulate* composites, dominated by annihilation from both positron- and Ps-related states, the unconstrained x3-term decomposed PAL spectra can be interpreted with different mathematical algorithms based on the canonical simple trapping model (STM) [16,17,18,19,20]. However, in a strong physical relevance, the STM is suitable for unconstrained two-component PAL spectra in solids having only one kind of positron trap in overall positron–electron annihilation (where annihilation from Ps-related sites is negligible). Therefore, the multi-channel PAL spectra of nanosubstances having a great variety of FVE are to be processed with STM, modified for some close-to-realistic restrictions.

#### 2.3.1. Canonical Two-State STM Ignoring Ps-Decaying

One of the most simplified approaches used to analyze unconstrained three-component PAL spectra in nanosubstances is canonical two-state STM [16,17,18,19,20] applied under conditions of slight input from the Ps-decay channel. This model describes positron (not Ps) annihilation from one kind of FVE, such as nm-sized atomic/sub-atomic voids. Being trapped by such structural defects, the positron annihilates with the *τ*_2_ = *τ_d_* lifetime approaching ~(0.2–0.5) ns [18,19]. Under a small Ps-decay contribution, the positron trapping channel is balanced by input from delocalized positrons annihilating directly from defect-free bulk states, obeying a two-state trapping scheme with a compensating (*τ*_1_,*I*_1_) component and average lifetime for trapped positrons *τ_av_^tr^.* defined as

(2) $$\tau_{av}^{tr.}=\eta_{b}\cdot \tau_{b}+\eta_{d}\cdot \tau_{d}=\tau_{1}\cdot I_{1}+\tau_{2}\cdot I_{2}$$

where *η_d_* =(1 − *η_b_*) and *η_b_* are fractions of defect-trapped and free annihilated positrons [19].

Thus, within two-state STM [16,17,18,19,20], the shortest *τ*_1_ lifetime is only the reduced bulk lifetime, which is connected (but not equivalent) to the defect-free bulk positron lifetime *τ_b_*:

(3) $$\tau_{b}=\frac{\tau_{1}\tau_{2}}{I_{1}\tau_{2}+I_{2}\tau_{1}}$$

In this case, the positron-trapping rate in defects *κ_d_* and the fraction of trapped positrons *η_d_* can be simply calculated as

(4) $$\kappa_{d}=\frac{I_{2}}{I_{1}}\left(\frac{1}{\tau_{b}}-\frac{1}{\tau_{2}}\right)$$

(5) $$\eta_{d}=\frac{\kappa_{d}\cdot \tau_{b}}{1+\kappa_{d}\cdot \tau_{b}}=\tau_{1}\cdot \kappa_{d}$$

Apart from above parameters, the difference between defect-specific and defect-free bulk lifetimes (*τ*_2_ – *τ_b_*) is accepted as the size signature of positron-trapping sites in terms of the equivalent number of vacancies, whereas the ratio between lifetimes (*τ*_2_/*τ_b_*) is ascribed to the nature of these traps [18].

In substances affected by nanostructurization, the evolution processes related to positron trapping-responsible FVE could be identified by employing the fitting parameters and trapping modes derived from the two-state STM. Thus, the trapping rate *κ_d_* occurs to be defect dependent:

(6) $$\kappa_{d}=C\cdot \mu$$

where *C* is the defect concentration and *μ* is the positron trapping coefficient (which approaches ~10^15^ atom·s^−1^ for negative vacancies in elemental and compound semiconductors [18]).

**Figure 1:**
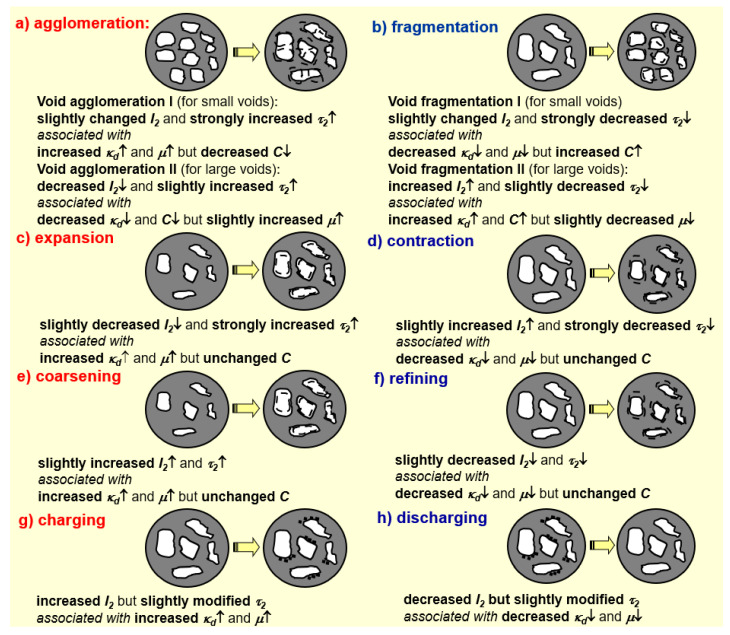
Schematic cartoons showing a variety of void-modification processes associated with PAL-detectable changes in positron-trapping modes ascribed to FVE under nanostructurization by preferential changes in volume ((**a**)—agglomeration, (**b**)—fragmentation, (**c**)—expansion, (**d**)—contraction) and environment ((**e**)—coarsening, (**f**)—refining, (**g**)—charging, (**h**)—discharging). Dashed counter lines correspond to sizes of positron-trapping FVE before nanostructurization.

**Positron trapping-modification processes** in nanostructurized substances are governed by changes in the number of positron traps, as well as in their volume and chemical environments, being principally different for small and large FVE [18,19,20]. A strong increase in the trapping coefficient *μ* is expected with the size for small vacancies, while trapping is close to saturation for large vacancies and vacancy-like clusters where the trapping rate *κ_d_* becomes strongly dependent on defect concentration *C* [18,30]. Typical views of main modification processes in FVE, responsible for positron trapping in nanostructured substances governed by deviations in their volume and environment, are reproduced schematically in Figure 1a–d.

Under ***agglomeration*** (Figure 1a) [20,31], voids get favorable environments to grow in size due to their merging (so one can expect a decrease in the number of voids accompanied by increase in their size). In case of small voids involving no more than several vacancies (referred to as *agglomeration I*), a strong increase in *τ_d_* = *τ*_2_ (and *μ*) leads to an increased trapping rate *κ_d_* despite a decrease in void concentration *C*. As it follows from Equation (4), these changes agree with a slight deviation in the *I*_2_ intensity. In the case of large voids (*agglomeration II*), a slight increase in the *τ_d_* = *τ*_2_ lifetime is expected. Thus, the trapping rate *κ_d_* drops down, owing to a preferential decrease in *C* and a slight increase in *μ*. Consequently, the *I*_2_ intensity decreases, as it follows from Equation (4). In an opposite case of ***void fragmentation***, reproduced in Figure 1b [20], voids have an unfavorable environment to grow in, and tend to be tiny in size due to the dividing of a few distinct parts. For small voids (*fragmentation I*), one can expect a strong decrease in the *τ_d_* = *τ*_2_ lifetime and trapping rate *κ_d_* (with respect to a preferential decrease in the trapping coefficient *μ* and an increased number of voids *C*), resulting in slight deviations in *I*_2_. For large voids (*fragmentation II*), a decrease in *τ_d_* is accompanied by increased *κ_d_* (due to slightly decreased *μ*), resulting from increased *I*_2_.

Under ***void expansion***, FVE have favorable environments to grow in size without essential changes in concentration *C* [20] (Figure 1c). These changes are concomitant with increased positron trapping efficiency of voids due to excess of free volume. The expanded voids are characterized by increased *τ_d_* = *τ*_2_ and *κ_d_*, but these increments are undetectable for large FVE, having slightly increased *μ*. Under such conditions, an expected trend in *I*_2_ is ambiguous, dominated by slightly decreased *I*_2_ for nanocomposites. By analogy, ***void contraction*** (Figure 1d) [20] is considered as the process getting FVE environments, suppressing their sizes without changes in void concentration C, the expected changes in positron-trapping modes being opposite to those caused by void expansion.

Schematic presentation of deviation processes occurring in positron trapping-responsible FVE in nanosubstances governed by changes in void environments is shown in Figure 1e–h. Under ***void coarsening*** (Figure 1e) [20,31], FVE get favorable environments for positron trapping due to the excess of free volume at the boundary surface (also without changes in *C*). This process is revealed by slightly increased *τ_d_* = *τ*_2_ and *I*_2_ associated with increased *κ_d_* and *μ.* By analogy, under ***void refining*** [20], FVE get unfavorable environments for positron trapping due to reduced boundary free volume (voids get more refined, but thinner in their boundaries, see Figure 1f).

For the sake of completeness, we also consider ***void charging*** and ***void discharging*** processes, shown respectively in Figure 1g,h [20]. The void charging supposes the appearance of the positron-attractive negative charge near FVE enhancing trapping efficiency. Under void discharging, the negative charge near FVE is lost by attaining a positron-repulsive neutral or more positive charge. Void charging causes slightly modified *τ_d_* = *τ*_2_ due to changes in the annihilation rate of more negatively-charged site, but substantially increased *κ_d_* and *μ*. In respect to Equation (4), such changes lead to increased *I*_2_. The process of void discharging is characterized by opposite changes.

Noteworthy, strict differentiation in the above void-modification processes for nm-scaled nanostructurization-driven volumetric effects is quite ambiguous in view of similarity between expected PAL-detectable responses derived, assuming unchangeable contributions in the defect-free bulk positron annihilation channel. Nevertheless, in combination with other complementary research employing conventional atomic-specific probes (such as X-ray diffraction analysis), the realistic nature of respective nanostructural transformations can be clarified.

Specifically, canonical two-state STM [16,17,18,19,20] describes positrons annihilating from two distinct sources, these being lattice-specific and defect-localized states ignoring the back escape of trapped positrons [16,17,18,19,20]. The remaining input in the reconstructed PAL spectra is caused by **Ps-decay contribution** due to positrons annihilating from Ps-related FVE (holes, voids, pores) as free particles, or interacting with electrons from the environment [16,18,24]. In a ground state, the Ps atom exists as para-Ps (p-Ps, antiparallel positron–electron spins) decaying intrinsically with two γ-quanta and 0.125 ns lifetime (in a vacuum), and ortho-Ps (o-Ps, having parallel configuration of positron–electron spins) decaying with three γ-quanta and 142 ns lifetime, these states being occupied with 1:3 probability. In a matter, since the positron wave function overlaps with the electron outside, annihilation with such electron from the environment having antiparallel spin decreases the o-Ps lifetime to 0.5–10 ns, resulting in two γ-rays (the “pick-off” annihilation) [18,24]. Two conditions should be satisfied to form the Ps-decaying state in solid, the first being sufficiently *great free volume* available to capture the bound positron–electron system, and the second being *low electron density* preventing the direct annihilation [24,32]. This specificity defines selective distribution of the positron and Ps traps in nanostructured media.

The Ps atoms localized in free-volume holes of the substance gives indication on the mean radii *R* in terms of the long-lived *τ*_3_ lifetime (the *I*_3_ value correlates with the density of the Ps-decay sites), with respect to the semi-empirical Tao–Eldrup equation:

(7) $$\tau_{3}=0.5\cdot {\left[1-\frac{R}{R+\Delta R}+\frac{1}{2\pi }\cdot \sin\left(\frac{2\pi R}{R+\Delta R}\right)\right]}^{-1}$$

where Δ*R* = 0.166 nm is the fitted empirical layer thickness [24].

By fitting the above Equation (7) with the measured longer-lived *τ*_3_ lifetime, the *R*_3_ radius and respective FVE *V_f_* (in spherical approximation) can be determined. The fractional free volume (*f_v_*) in the substance under consideration where Ps-related states are stabilized can be calculated as

*f_v_* = *C*’·*I*_3_·*V_f_* (8)

using empirical constant *C*’ = 0.0018 Å^−3^ validated for materials without structural groups inhibiting Ps formation (such as epoxy polymers [26]).

#### 2.3.2. Three-State Additive (Mixed Positron-Ps) STM

In case of stronger Ps-decaying yield, as in nanostructured substances [21,22,23,24,33,34,35,36,37,38], the collected x3-term decomposed PAL spectra reconstructed under constraint-free fitting can be analyzed in terms of the **three-state positron-Ps additive STM** assuming two inputs in an overall trapping process arising from trapped positrons and Ps-decaying states [39,40,41]. In fact, this model includes two distinct positron-trapping sources with different rates, these being *κ_d_*_1_ (for trapping from free positrons), and *κ_d_*_2_ (for trapping from Ps-decaying source):

(9) $$\kappa_{d1}=I_{2}\left(\frac{1}{\tau_{1}}-\frac{1}{\tau_{2}}\right)$$

(10) $$\kappa_{d2}=I_{3}\left(\frac{1}{\tau_{1}}-\frac{1}{\tau_{3}}\right)$$

allowing more correct estimation of the defect-free bulk positron lifetime *τ_B_*^1–2^ related to positron annihilation from the Bloch states [38]:

(11) $$\tau_{B}^{1-2}={\left(\frac{I_{1}}{\tau_{1}}+\frac{I_{2}}{\tau_{2}}+\frac{I_{3}}{\tau_{3}}\right)}^{-1}$$

Such approach is especially useful when dealing with comparative studies on different FVE in solids undergoing continuous changes in atomic-deficient structures under nanostructurization.

#### 2.3.3. Two-State Ps-Positron Trapping-Conversion Model (TCM)

In substances obeying unconstrained three-term decomposed PAL spectra with essential contributions in the third channel (*I*_3_), which cannot be recompensated due to correction in a source contribution, two boundary cases related to annihilation from Ps-decaying sites can be roughly distinguished in terms of fitting parameters only [42]. Thus, the prevailing trend in the Ps-formation probability (decrease or increase) would result in isotypical simultaneous changes in both *I*_1_ and *I*_3_ intensities accompanied by an opposite change in *I*_2_ intensity. In contrast, the changing trend in the hole density prevails, when simultaneous unison change in both *I*_2_ and *I*_3_ intensities are counterbalanced by an opposite change in *I*_1_ intensity.

In highly heterogeneous materials, positron annihilation occurs through mixed positron-trapping and Ps-decaying channels, obeying the concept of selective localization of respective trapping sites [32,43]. The low-electron density holes with maximum free volume and minimum surface tension should fit to confine Ps stabilization, owing to its repulsive exchange potential, while the regions of higher electron density (negatively charged) and polarization (such as sub-nm-scaled voids) are more suitable to capture electrically charged positrons (e^+^).

Therefore, for many nanosubstances, the positron–electron (e^+^e^−^) annihilation is expected through interconnected positron-Ps related channels, so that the Ps-decay sites are the only FVE, which can be converted under nanostructurization into positron-trapping sites (and vice versa). In this case, the formalism of generalized two-state STM modified for Ps-to-positron trapping conversion, referred to as **x3-x2-CDA (coupling decomposition algorithm)** [21,22,23], is a powerful tool used to identify nanostructurization-driven atomic-deficient volumetric effects.

**Figure 2:**
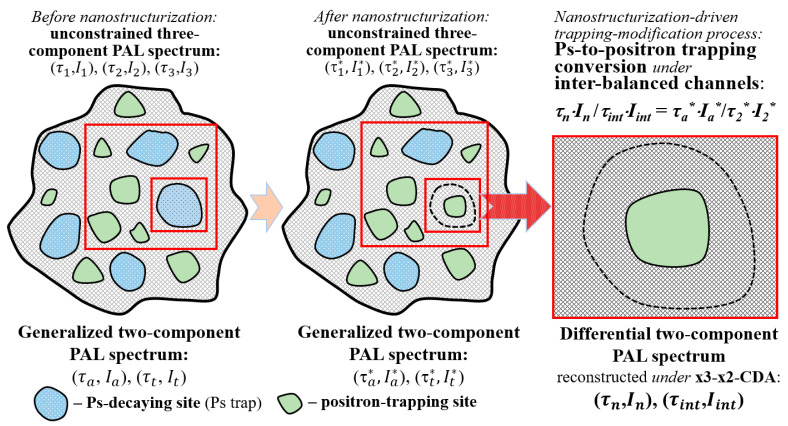
Fragment of atomic-deficient microstructure of substance under nanostructurization obeying completely inter-balanced Ps-to-positron trapping conversion in terms of x3-x2-CDA. Nanostructurization is revealed as the transformation of the Ps-decaying hole (blue-color, shown by the counter-dashed line after nanostructurization) into the positron-trapping site (green-color).

The action of this algorithm is exemplified schematically in Figure 2, showing transformation of the distinct Ps-decay site (blue-colored hole), in the positron trap (green-colored void), in the substance subjected to nanostructurization (which is revealed as complete conversion of the Ps-decaying hole into the positron-trapping void). The similar trapping-modification processes can be realized due to partial occupation of free volumes ascribed to Ps-related holes by some *nanostructurizing* elements (the known problem of *host–guest* chemistry dealing with filling of open nanospaces in the *host* matrix by *guest* entities, such as atoms, molecules, atomic clusters, NP, etc. [23,44,45]). These processes reveal complete conversion from Ps-decay to positron-trapping channels in the PAL spectra, occurring at the same FVE (nominated as *tailoring-nanospace* problems [45]). Trapping-conversion processes are characters for *polymer/filler nanocomposites* (when filler NP are embedded into atomic-accessible free-volume *holes* of the polymeric matrix, operational as effective Ps-decay sites) [22,23,44,45]. Such changes can also be caused by defect formation in *mechanochemistry* [8,9,10,11], pressure-induced transformations, and interdiffusion processes in *multi-NP systems* [45,46], etc. In the above cases, one deals with substances possessing unconstrained three-component PAL spectra, nanostructurized by transforming initial Ps-decaying holes into free positron self-annihilation sub-nm-scaled voids without contributions from other competitive channels. Within this approach, the straightforward parameterization of nanostructurization-driven volumetric effects can be developed using the modified STM, describing the transition from the three-component to the generalized two-component PAL spectra.

Firstly, this analysis is to be applied to the *unstructured* matrix having (*τ*_1_*,I*_1_), (*τ*_2_*,I*_2_), (*τ*_3_*,I*_3_) inputs in unconstrained x3-term decomposed PAL spectrum obeying normalization *I*_1_ + *I*_2_ + *I*_3_ = 1 (see Figure 2).

This three-component PAL spectrum can be transferred to the generalized two-component form by removing inputs from p-Ps annihilation (with *τ*_p_ = 0.125 ns lifetime and *I*_p_ = *I*_3_*/3* intensity) from the first channel and o-Ps “pick-up” annihilation (with *τ_3_* lifetime and *I*_3_ intensity) from the third channel to the general trapping channel. Thus, we can simply estimate contribution (*τ_a_*,*I_a_*) to the first channel other than p-Ps:

*τ_a_*·*I_a_* = *τ*_1_·*I*_1_ − *τ_p_*·*I_p_* (12)

*I_a_* = *I_1_* − *I_p_* (13)

**Figure 3:**
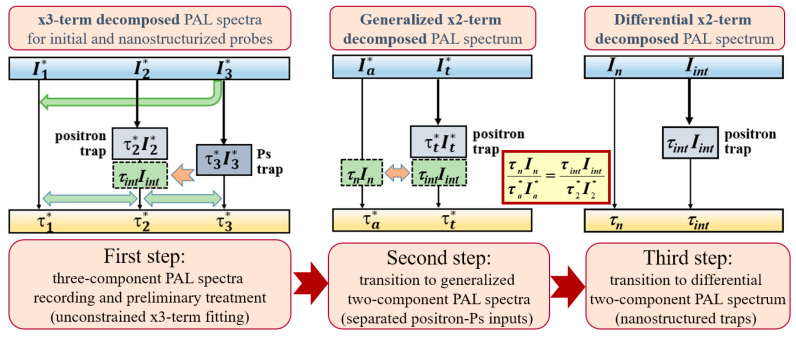
Schematic presentation of algorithm parameterizing differential two-component PAL spectrum of nanosubstance obeying Ps-to-positron trapping conversion, in terms of x3-x2-CDA. The unconstrained three-component PAL spectra of initial and nanostructurized substances are first collected, and transformed to the generalized two-component form, assuming completely inter-balanced positron-Ps contributions. Differential two-component PAL spectrum with “pure” nanostructurization-related contributions (*τ_n_*,*I_n_*) and (*τ_i_*_nt_,*I_int_*) is reconstructed.

The similar calculations should be performed for *nanostructurized* substance having (*τ*_1_*^∗^,I*_1_^∗^), (*τ*_2_*^∗^,I*_2_^∗^), (*τ*_3_*^∗^,I*_3_^∗^) inputs in the unconstrained three-component PAL spectrum (normalized by intensities *I*_1_^∗^ + *I*_2_^∗^ + *I*_3_^∗^ = 1), assuming the second component in the generalized PAL spectrum, as in the initial *unstructured* matrix (see Figure 3):

*I*_2_^∗^ = *I_int_* + *I*_3_^∗^·(*I*_2_/*I*_3_) (14)

*τ*_2_^∗^·*I*_2_^∗^ = *τ_int_*·*I_int_* + *τ*_2_·(*I*_2_^∗^ − *I_int_*) (15)

Nanostructurization-driven Ps-to-positron trapping conversion can be refined by the transition to the *differential two-component PAL spectrum* with first and second component inputs defined as

*τ_n_*·*I_n_* = *τ_a_*^∗^·*I_a_*^∗^ − *τ_a_*·(*I_a_*^∗^ − *I_n_*,) (16)

*τ_int_*·*I_int_* = *τ_2_*^∗^·*I*_2_^∗^ − *τ*_2_·(*I*_2_^∗^ − *I_int_*) (17)

assuming full inter-channel balance in the substance under nanostructurization (the contributions from derived components are equilibrated with those from which these components arise [21]):

*τ_n_*·*I_n_*/*τ_int_*·*I_int_* = *τ_a_*^∗^·*I_a_*^∗^ /*τ_2_*^∗^·*I_2_*^∗^ (18)

Complete parameterization of Ps-to-positron trapping conversion in the nanostructurized matrix (as shown in Figure 2 and Figure 3) is performed, accepting (*τ_n_*, *I_n_*) and (*τ_i_*_nt_, *I_int_*) as the respective first and second components of the reconstructed differential PAL spectrum, employing formalism of conventional two-state STM [16,17,18,19,20].

Within this approach, the preferential directionality in the trapping conversion is defined by the sign of both (*I_n_* and *I_int_*) intensities (if both intensities are positive, the direct Ps-to-positron trapping conversion occurs; while if both intensities are negative, the inverse positron-to-Ps trapping conversion occurs) [21,22,23]. Trapping modes derived from **the Ps-positron trapping-conversion model** in accord to the above Equations (2)–(5) are signatures of the nanostructured (NP-modified) matrix, involving both Ps-decay and positron-trapping related sites, these being defect-specific *τ_int_* and defect-free bulk *τ_b_^NP^* lifetimes, trapping rate in defects *κ_d_^NP^*, and some derivative characteristics, relevant to the trap size, in terms of the equivalent number of vacancies defined by (*τ_int_* − *τ_b_^NP^*) difference and nature of these traps, defined by *τ_int_/τ_b_^NP^* ratio.

In contrast to the free-constraint x3-term decomposition parameterizing the PAL spectra, in terms of simple *uncorrelated positron-Ps trapping*, the x3-x2-CDA allows identification of separated contributions in the nanostructurized substance. However, this approach becomes meaningless, if competitive contributions from other trapping-modification processes are possible under nanostructurization (e.g., changes in annihilation from delocalized Bloch states, or changes in the population of other FVE responsible for positron and/or Ps-trapping). Furthermore, this approach is inefficient, if both (*τ_n_*,*I_n_*) and (*τ_int_*,*I_int_*) components are unsaturated under nanostructurization, allowing only negligible contributions in the overall balance of annihilation events. Thereby, the x3-x2-CDA can be used as indicative, separating processes of nanostructurization-driven host matrix modification from the simple interplay between distinct uncorrelated positron-trapping and Ps-decaying channels.

## 3. Results and Discussion

**Figure 4:**
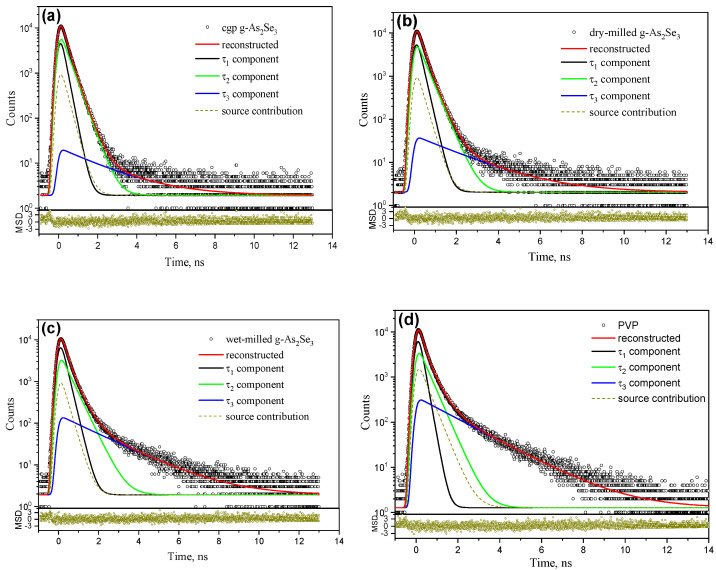
The PAL spectra of pelletized unmilled (**a**), dry-milled (**b**), and wet-milled g-As_2_Se_3_ (**c**) reconstructed from unconstrained x3-term fitting at the general background of the source contribution. The PAL spectrum of PVP pellets reconstructed under the same decomposition using 2 M of accumulated annihilation events [47] is shown for comparison (**d**). The bottom inset represents the statistical scatter of variance in favor of good applicability of the applied fitting.

In the current research, the collected PAL spectra of pelletized arsenoselenides As_x_Se_100−x_ were adequately reconstructed with an unconstrained x3-term decomposition routine. As an example, three-component PAL spectra of cgp (unmilled) and fgp (dry-milled and wet-milled) pellets of g-As_2_Se_3_ are reproduced at the general background of the source contribution in Figure 4.

Good applicability of this fitting with three unconstrained components is proved by the statistical scatter of variance (minimal statistically weighted least-square deviation between the experimental and theoretical curve built of three exponentials) tightly grouped along the time axis. For comparison, the PAL spectrum of “pure” PVP pellets reconstructed under the same routine using 2 M of annihilation events taken from our preliminary research [47] is also reproduced in Figure 4d. The respective PAL spectra fitting parameters for pelletized g-As_x_Se_100−x_ and PVP are presented in Table 1.

### 3.1. Volumetric Nanostructurization in g-As_x_Se_100−x_ under Modified Two-State STM

Within a conventional case involving mixed positron-Ps annihilation channels, an adequate resolution exists only for some close-to-boundary restrictions ignoring either Ps-decaying or positron-trapping contributions. The latter are more realistic, at least, for cgp and fgp dry-milled pellets having a low third component *I*_3_ intensity (Table 1).

The trapping modes derived from unconstrained x3-term decomposed PAL spectra of pelletized unmilled, dry-milled, and wet-milled g-As_x_Se_100−x_, assuming two-state STM with ignored Ps-decay contribution are given in Table 2 (for comparison, the data for PVP pellets derived under the same decomposition route [47] are also included).

Three-component spectrum of pelletized unmilled (cgp) arsenic triselenide g-As_2_Se_3_ reveals a second component with a defect-specific positron lifetime *τ*_2_ ~ 0.358 ns (see Table 1), which is at the character level for triple/quadruple atomic vacancies in binary g-As-Se [48,49,50,51,52,53]. The third component with relatively small fractional free volume *f_v_* = 0.15% and long-lived lifetime *τ*_3_ ~ 2.091 ns can be ascribed to Ps-related holes with radius approaching *R* ~ 0.296 nm. Despite a small concentration of these holes (since *I*_3_ intensity is less than 1%), this component cannot be eliminated from consideration without substantial reduction in the fitting goodness. Nevertheless, we also decomposed PAL spectrum in (evidently worse) two distinct components, to compare these data with those for the same bulk g-As_2_Se_3_ treated within two-state STM [50]. The revealed excellent coincidence in defect-specific *τ_d_* = *τ*_2_ and defect-free *τ_b_* positron lifetimes, as well as trapping rate in defects *κ_d_*, speaks in a favor of tight proximity between these samples.

The most simplified insight on nanostructurization-driven volumetric changes in g-As-Se can be done in terms of the average positron lifetime *τ_av_.* (1) derived from canonical STM.

In the batch of **pelletized unmilled g-As_x_Se_100−x_**, with deviation from stoichiometry As_2_Se_3_ towards Se-rich compositions, the *τ_av_.* lifetime derived from three-component PAL spectra (0.309 ns) decreases to ~0.285 ns, while demonstrating slow increase to ~0.32 ns with approaching As-rich samples (see Table 1). The similar behavior (albeit without detectable changes for As-rich probes) is character for *τ_av_^tr^.* lifetime (2), calculated for trapped positrons within STM, ignoring Ps-decay contribution (see Table 2). This compositional trend (excepting over-stoichiometric arsenoselenides) is concomitant with a sharp increase in the *τ_av_.* lifetime for bulk g-As-Se obtained, by quenching from a melt approaching g-As_2_Se_3_ [54], where trapping occurs in intrinsic sub-nm-scaled FVE, such as agglomerates of bond-free solid angles allocated at Se atoms in the bottom of the interlinked AsSe_3/2_ pyramidal units [49,50,51,55]. More moderated behavior of *τ_av_.* in these glasses above As_2_Se_3_ composition is caused by stabilization of molecular-network conformations dominated by realgar-type As_4_Se_4_ units [56]. From this analysis, it is clear that additional positron trapping sites are generated in over-stoichiometric g-As_x_Se_100−x_ (x > 40) under coarse powdering, followed by the pelletization procedure.

In the batch of **pelletized fgp dry-milled g-As_x_Se_100−x_**, compositional dependence of both average lifetimes *τ_av_.* (1) and *τ_av_^tr^.* (2) do not change substantially (see Table 1 and Table 2), but show somewhat lower values for under-stoichiometric samples (x < 40), and higher values for over-stoichiometric samples (x ≥ 40). This specificity is connected with a recently discovered enhanced milling effect on molecular confirmations in this system [57,58].

Neglecting o-Ps-decaying contributions in the reconstructed PAL spectra (believed to be quite reasonable for both unmilled and dry-milled g-As_x_Se_100−x_ in view of *I*_3_ < 1.5%), it is clear that slight agglomeration of large positron traps (the process of void agglomeration II depicted in Figure 1a) is expected in transition from unmilled to dry-milled arsenoselenides. Indeed, under this transition, a decrease in *I*_2_ intensity is accompanied by a slight increase in *τ*_2_ lifetime (Table 1), resulting in a merely depressed positron trapping rate in defects *κ_d_* (Table 2). The only exception can be found at the edges of the glass-forming region in g-As_x_Se_100−x_ system (for x = 5, 10, 55, 65).

The most prominent changes are found in the batch of **pelletized fgp wet-milled g-As_x_Se_100−x_**. In these g-As_x_Se_100−x_/PVP nanocomposites, the average lifetimes for trapped positrons *τ_av_^tr^.* are slightly enriched on ~0.02–0.03 ns in respect to the same values in unmilled samples (Table 2), while average lifetimes derived from three-component PAL spectra *τ_av_.* (1) demonstrate a sharp ~0.07–0.08 ns increment for over-stoichiometric As-rich probes, and ~0.15–0.19 ns increment for under-stoichiometric Se-rich probes (Table 1). Thus, in g-As_60_Se_40_/PVP and g-As_5_Se_95_/PVP nanocomposites, the *τ_av_.* approaches 0.466 ns and 0.472 ns, the values far beyond *τ_av_*. ~0.386 ns detected for stoichiometric g-As_40_Se_60_/PVP. As a result, compositional dependence of *τ_av_.* is changed drastically, attaining broad minimum near PVP-capped g-As_2_Se_3_. Of course, because of a strong contribution from o-Ps decaying in the reconstructed PAL spectra (Figure 4c), their analysis, in terms of alone positron trapping modes [20], is impossible.

It is worth mentioning, in the studied As_x_Se_100−x_/PVP nanocomposites, the longest lifetime *τ*_3_ is dropped to ~1.7–1.9 ns, tending to o-Ps-related lifetime in “pure” PVP (*τ*_3_ = 1.867 ns) [47]. This means that Ps atoms are stabilized rather in the PVP environment than in g-As-Se matrix, their decay being substantially enhanced in the fractional free volumes *f_v_* (see Table 2).

Thereby, in comparison with unmilled (cgp) and dry-milled glassy arsenoselenides, positron trapping in PVP-capped g-As_x_Se_100−x_ (wet-milled probes) is modified because of enriched Ps-decaying states stabilized in a preferentially “pure” PVP environment.

In inhomogeneous multi-particulate substances composed of agglomerated NP, the large portion of positrons thermalized inside grains travel towards GB and interparticle TJ (since their diffusion lengths become comparable with grain size), and annihilate after getting trapped into these trapping sites. Because of a large amount of FVE generated at NM [59,60,61], this specificity determines the complication in the PAL spectra collected for fgp (dry-milled and wet-milled) samples as compared with cgp (unmilled) samples.

The PAL spectra of pelletized dry-milled g-As_2_Se_3_ demonstrates increased *τ*_2_~0.371 ns as compared with this value in the unmilled probe, but rather unchanged Ps-related *τ*_3_~2.087 ns lifetime (Table 1). Meanwhile, the average positron lifetime *τ_av_* is only slightly increased in this sample under NM, the similar tendencies being observed in other arsenoselenides with average deviation of ±0.01 ns. The relative stability of *τ_av_.* lifetime in these probes is counterbalanced by decreased input from the second component. Thus, the *I*_3_ intensity is unchanged or even slightly increased in dry-milled samples (see Table 1), resulting in equivalent growth (albeit not strongly evident) in fractional free volume *f_v_* (see Table 2).

Assuming two inputs contributing to an overall trapping in the studied glassy g-As-Se arising from distinct positron- and Ps-related traps, the reconstructed PAL spectra can be adequately described in terms of two-state STM expanded to accommodate second defect-related component instead of Ps-decay one (**three-state additive STM**) as was done in [39,40,41]. This approach should not be taken too seriously in view of typical resolution in reconstructed PAL spectra [39], but it is useful to analyze bulk positron lifetimes and compositional behavior of trapping rates in multichannel positron annihilation process [41]. The trapping modes for pelletized g-As_x_Se_100−x_ specimens (unmilled, dry-milled, and wet-milled ones) calculated in terms of three-state additive STM are given in Table 3.

Two types of principally different traps can be distinguished in the studied nanocomposites, these being short- and long-lived ones with *κ_d_*_1_ and *κ_d_*_2_ trapping rates, respectively (*κ_d_*_1_ > *κ_d_*_2_). In transition from unmilled to dry-milled probes, the latter are nearly unchangeable, possessing the *κ_d_*_2_ rate at the level of ~0.05–0.07 ns^−1^. Under this transition, the former traps show a clearly decreasing tendency in their trapping rate *κ_d_*_1_ ascribed to the process of void agglomeration II schematically illustrated in Figure 1a (apart from compositions at the edges of the glass-forming region). The defect-free bulk positron lifetime *τ_B_*^1–2^ in these probes approaches ~0.25–0.27 ns, confirming that annihilating positrons are trapped in intrinsic FVE stabilized in g-As-Se environment [48,49,50,51,52,53].

Under transition to wet-milled arsenoselenides, the role of long-lived positron trapping sites is enormously enhanced so that their trapping rate *κ_d_*_2_ becomes only 2–5 times less as *κ_d_*_1_ (see Table 3). The defect-free bulk positron lifetime *τ_B_*^1–2^ is also enriched in the pelletized samples of g-As_x_Se_100−x_/PVP nanocomposites to approximately ~0.28–0.29 ns. Hence, the medium where positron annihilation occurs is cardinally changed in wet-milled glassy arsenoselenides, being transferred rather to the PVP solution.

### 3.2. Volumetric Nanostructurization in Dry-Milled and Wet-Milled g-As_x_Se_100−x_ in Terms of Ps-to-Positron Trapping Conversion (x3-x2-CDA)

If processes of Ps-decaying and positron {e^+^}-trapping are interconnected so that no changes occur in other alternative channels, unconstrained three-term decomposed PAL spectra can be treated employing the formalism of recently developed x3-x2-CDA [21,22,23]. With this in mind, let us reconstruct the expected and most plausible positron annihilation channels in the studied g-As_x_Se_100−x_-based nanocomposites using the respective matrix shown in Table 4.

**Annihilation from defect-free bulk (DFB) states** is assumed to be the most relevant and completely invariant process in all glassy arsenoselenides g-As-Se, whatever their technology. In Table 4, this channel of an intrinsic positron annihilation is denoted as {DFB}^g-As-Se^–int for all groups of samples (bulk, cgp, and fgp; unmilled and milled; pelletized and unpelletized).

**Annihilation from positron (e^+^)-traps** (positron trapping) is an alternative path of free positrons used to annihilate with electrons through defective states acting as sites capturing positrons. One group of these (e^+^)-traps, denoted in Table 4 as {e^+^}^g-As-Se^–int, is connected with intrinsic sub-nm-scaled FVE proper to glassy arsenoselenides. The most effective positron trapping centers of this type are multiatomic vacancies stabilized in the g-As-Se network at the bottom of AsSe_3/2_ pyramids in a preferentially negative environment of chalcogen (Se) atoms [48,49,50,51,52,53]. Another group of positron traps (denoted in Table 4 as {e^+^}^g-As-Se^–IF-1,2,3) is character to fgp g-As-Se, these FVE being stabilized under pelletization [61] as GB and TJ (i.e., intersection between three or more adjoining NP) in samples of different milling, pre-history [62,63,64,65,66].

**Annihilation from Ps-decaying states** ascribed to nanostructured FVE (free-volume sub-nm-scaled holes, voids, pores, cavities, etc.) dominates in sub-nanometric porous structures by interaction with electrons from the environment, owing to the “pick-off” process [18,24,32]. One part of these defects (denoted in Table 4 as {Ps}^g-As-Se^–int-1,2) are connected with decaying of bound positron–electron states (Ps atoms) in intrinsic imperfections of a glassy state stabilized under rapid melt-quenching. It can be reasonably assumed that this source of Ps-decaying site (yellow-colored in Table 4) is completely analogous in all pelletized samples. Another part of the Ps-decaying site is related to FVE, marked in Table 4 as {Ps}^g-As-Se^–FVE-1,2,3, which appears specifically in the powdered glassy arsenoselenides owing to material attrition on the finest nanometer pieces. These FVE represent IF free-volume spaces between contacting NP (such as disclinations or TJ between adjoining NP), where both conditions stabilizing Ps-related states [24,32] are satisfied. For metallic multi-particulate systems, the most essential excess of additional free volume is expected with individual crystallites less than 5–10 nm [62,63,64,65].

Under this consideration, it is evident that *conversion* should be expected in the respective channels in dry-milled and wet-milled samples (grey-colored in Table 4), involving changes connected with positron trapping from {e^+^}^g-As-Se^–IF/GB-2 to {e^+^}^g-As-Se/PVP^–IF/GB-3 sites, and Ps-decaying from {Ps}^g-As-Se^–FVE-2 to {Ps}^g-As-Se/PVP^–FVE-3 sites.

The calculated trapping modes for dry-milled glassy arsenoselenides g-As_x_Se_100−x_, defined in respect to iso-compositional g-As_x_Se_100−x_/PVP nanocomposites, employing formalism of x3-x2-CDA [21,22,23], are gathered in Table 5.

Positive intensities of both *I_n_* and *I_int_* components in the generalized x2-component PAL spectra reconstructed from respective unconstrained x3-component spectra of pelletized dry-milled and wet-milled samples, along with well-defined component inputs (*τ_n_*·*I_n_*) and (*τ_int_*·*I_int_*), testify that {Ps}-decay sites in wet-milled samples can be imagined as directly transformed to {e^+^}-traps in dry-milled ones. The derived values of defect-related positron lifetimes at the level of *τ_int_* = 0.36–0.38 ns testify that {e^+^}-traps are extended IF defects, close in their volumes to di- and/or tri-atomic vacancies having ~0.25–0.28 nm in radius (stabilized at the bottom of AsSe_3/2_ pyramids in negative environment of Se atoms), as it follows from the known semi-empirical relationship for these glasses [51,52,53]. This finding agrees well with (*τ_int_* − *τ_b_^NP^*) = 0.09–0.11 ns and *τ_in_*_t_/*τ_b_^NP^* = 1.34–1.46 as indicatives of these vacancy-type defects [18]. Both parameters are slightly increased as going towards under-stoichiometric Se-rich glassy arsenoselenides g-As_x_Se_100−x_ along with decreased trapping rate in defects *κ_d_^NP^* (see Table 5), testifying that AsSe_3/2_ pyramids are destroyed by an abundant amount of homonuclear Se-Se covalent chemical bonds (causing void agglomeration II, as schematically depicted in Figure 1a).

In a particular case of dry-milled g-As_2_Se_3_, positron {e^+^}-trapping channel substitutes {Ps}-decaying contribution from empty inter-particulate junctions with *τ*_3_ = 2.087 ns by input from PVP-filled ones with *τ*_3_ = 1.891 ns (Table 5). The governing trapping-conversion process, i.e., appearance of positron {e^+^}-traps with defect-related lifetime *τ_int_* = 0.352 ns instead of Ps-decaying sites with *τ*_3_ = 2.087 ns, occurs in the environment possessing defect-free bulk lifetime *τ_b_^NP^* = 0.263 ns (Table 5). This value is evidently above the bulk lifetime in crystalline c-As_2_Se_3_ (*τ_b_* = 0.240 ns [47]), but below *τ_b_* in g-As_2_Se_3_ (~0.280–0.285 ns as determined from unconstrained x2-term decomposed PAL spectra ignoring input from Ps-decaying [50,51]). Thus, the chemical environment where Ps-to-positron trapping conversion occurs, i.e., inner spaces where {e^+^}-traps appear (in dry-milled g-As_2_Se_3_) instead of Ps-decaying sites (in PVP-capped g-As_2_Se_3_), is indeed glassy arsenoselenide matrix.

Concerning the x3-x2-CDA, we are grounded on the assumption that the process of Ps-to-positron trapping conversion is indeed the alone process linking dry-milled and wet-milled glassy arsenoselenides. This condition is satisfied, owing to nearly the same channel of positron–electron annihilation originating from DFB states in all g-As_x_Se_100−x_ samples (mainly, due to a small amount of PVP in wet-milled nanocomposites).

### 3.3. Positronics of Nanostructurization-Driven Volumetric Effects in g-As_x_Se_100−x_

**Figure 5:**
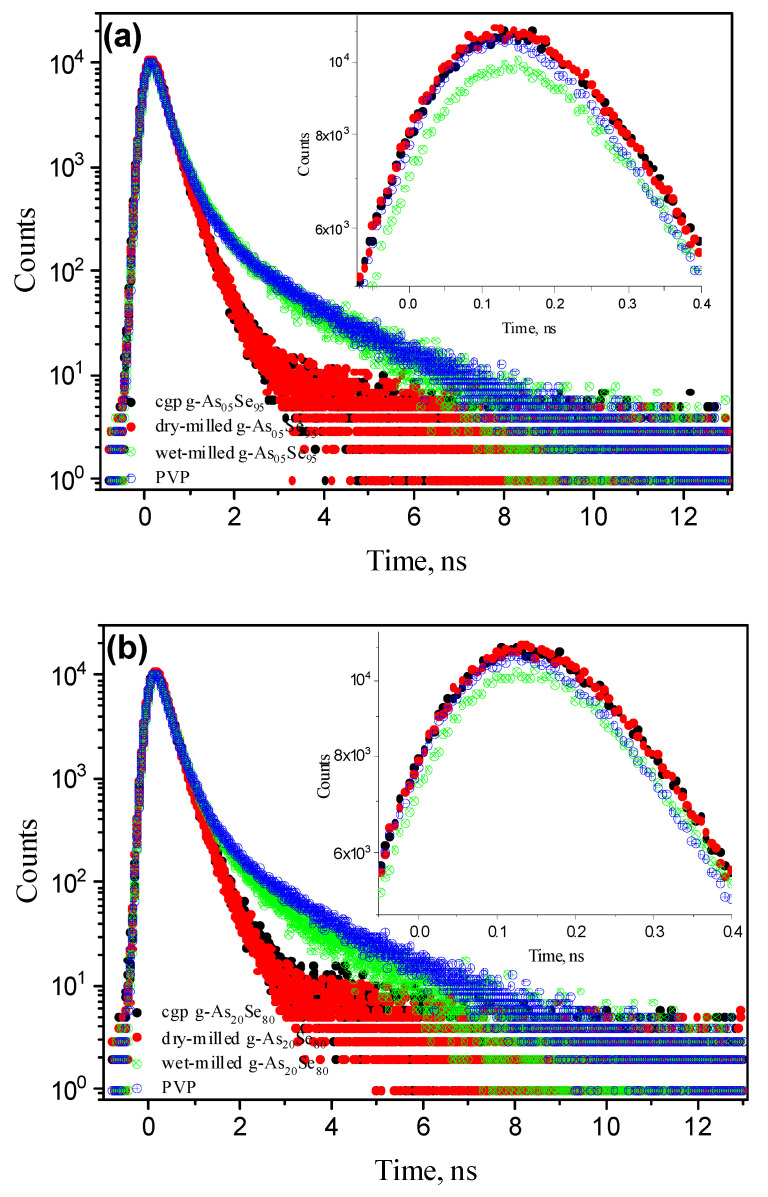
Nanostructurization-tuned “rainbow” effects in under-stoichiometric g-As_5_Se_95_ (**a**) and g-As_20_Se_80_ (**b**) pellets: the PAL spectra were collected for unmilled (black points), dry-milled (red points), and wet-milled glassy arsenoselenides (green points) and compared with PAL spectrum of PVP (blue points). The insert shows respective PAL spectra peaks of wet-milled probes, abruptly depressed in both wings and slightly shifted to higher lifetimes due to enhanced Ps-formation probability and increased mean positron lifetime *τ_av_*. The empty gap between PAL spectra “tails” for dry-milled and wet-milled samples is due to jump in hole density of o-Ps decaying states in transition to PVP medium.

**Figure 6:**
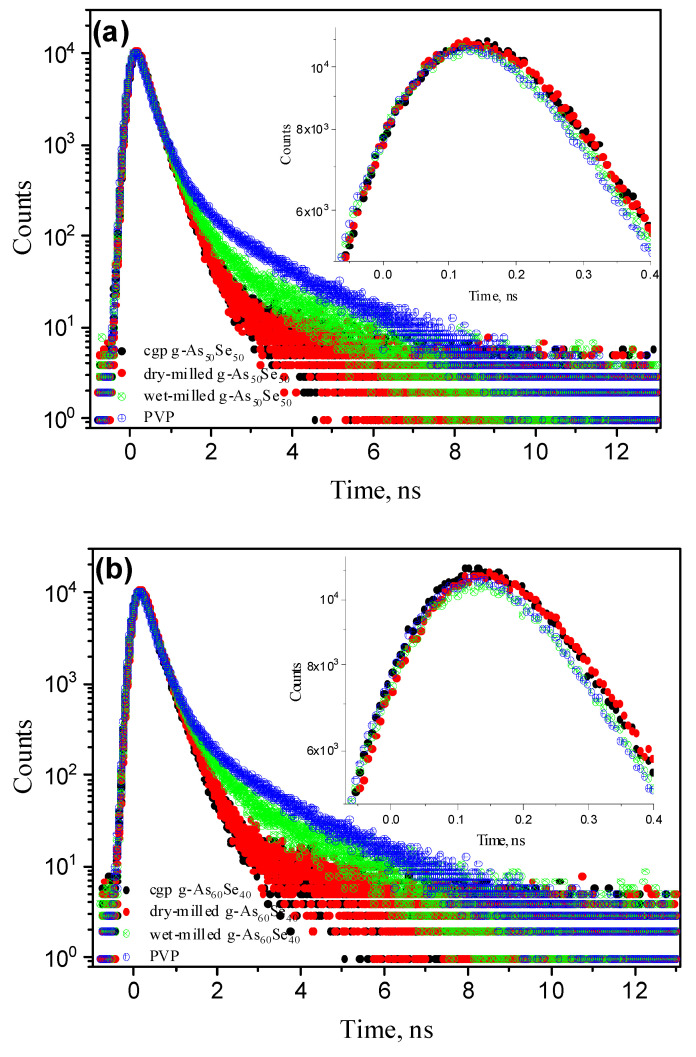
Nanostructurization-tuned “rainbow” effects in over-stoichiometric g-As_50_Se_50_ (**a**) and g-As_60_Se_40_ (**b**) pellets: the PAL spectra were collected for unmilled (black points), dry-milled (red points), and wet-milled glassy arsenoselenides (green points) and compared with the PAL spectrum of PVP (blue points). The insert shows nearly invariant tendency in the PAL spectra peaks depressed in the right wing after wet nanomilling due to moderated Ps-formation probability and slightly changed average positron lifetime *τ_av_*. Changes in the PAL spectra “tails” for unmilled, dry-milled, and wet-milled probes is owing to increase in the hole density of o-Ps decaying states. There is no empty gap between PAL spectra “tails” for dry-milled and wet-milled samples caused by changes in o-Ps decaying states in transition to the PVP medium.

Thus, the most striking feature of comparative PAL-spectra analysis in NM-modified glassy As-Se is an evidently decreasing trend in contribution to o-Ps decaying modes in the row of pelletized samples from PVP to wet-milled As_x_Se_100−x_, and further to iso-compositional dry-milled and unmilled specimens. Such variations with nanostructurally- and compositionally-tuned third-component contribution and average positron lifetime *τ_av_* compose an interesting specificity in *Positronics*, revealed in monotonic behavior of both PAL spectrum peaks and PAL spectrum “tails”, which can be highlighted as “rainbow” effects. A group of **nanostructurization-tuned “rainbow” effects** is spectacularly well revealed in reconstructed PAL spectra of studied As_x_Se_100−x_ (Figure 5 and Figure 6).

In over-stoichiometric g-As_50_Se_50_ and g-As_60_Se_40_ probes affected to NM, nanostructurization-tuned “rainbow” effects in the PAL spectra are revealed less specifically (Figure 6a,b). Owing to moderated Ps-formation probability and decreased average lifetime, these nanocomposites demonstrate a nearly invariant trend in the PAL spectrum peak, which is mainly depressed in the right wing after wet NM. Nevertheless, the PAL spectra “tails” are expanded in transition from unmilled and dry-milled specimens to PVP-capped ones without distinct empty gaps on the histogram of accumulated annihilation events (the changes in *f_v_* for dry-milled and wet-milled g-As_60_Se_40_ reach only ~0.67%, see Table 2).

**Figure 7:**
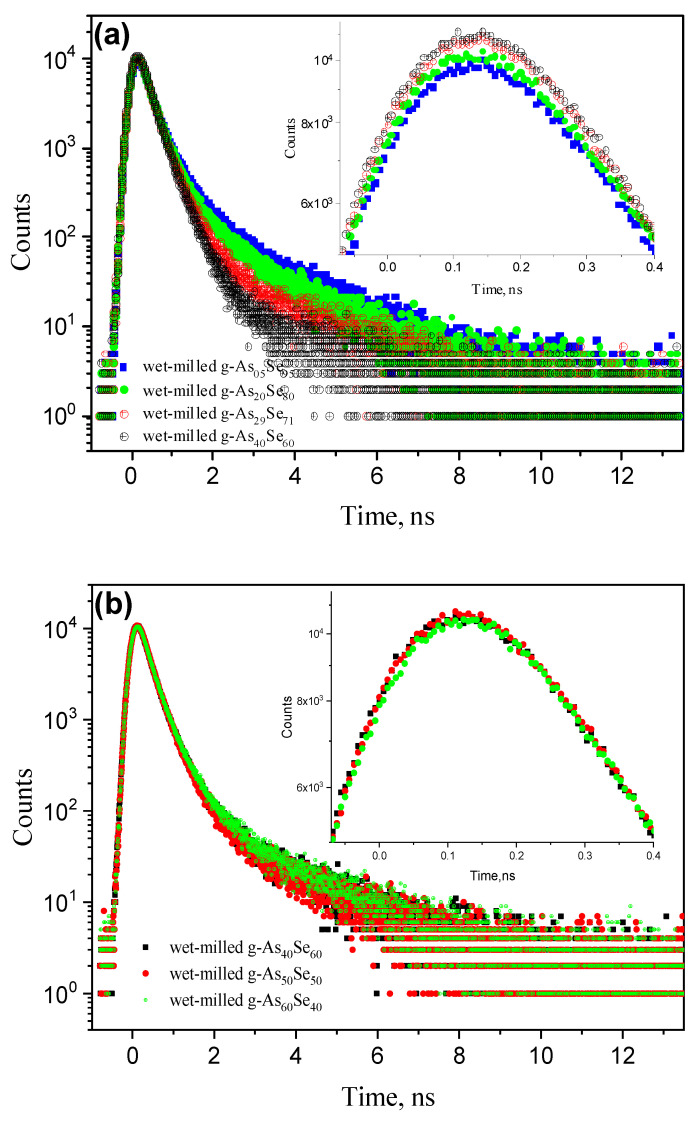
Compositionally tuned “rainbow” effects in PVP-capped g-As_x_Se_100−x_ pellets: (**a**)—under-stoichiometric samples of g-As_5_Se_95_ (blue points), g-As_20_Se_80_ (green points), g-As_29_Se_71_ (red points) and stoichiometric g-As_40_Se_60_ (black points); (**b**)—stoichiometric samples of g-As_40_Se_60_ (black points) and over-stoichiometric g-As_50_Se_50_ (red points) and g-As_60_Se_40_ (green point). The inserts show peak-changing trends in the PAL spectra of PVP-capped samples due to changes in average positron lifetime *τ_av_* and long-lived component intensity *I*_3_.

The group of **compositionally tuned “rainbow” effects** are detectable in the PAL spectra of g-As_x_Se_100−x_ affected to NM in a wet mode (viz. g-As_x_Se_100−x_/PVP nanocomposites) with a monotonically changed *x* parameter (see Figure 7).

These effects are more pronounced in under-stoichiometric arsenoselenides (*x* < 40, Figure 7a), demonstrating (with going to “pure” Se) a clearly depressing tendency in the PAL spectrum peak (symmetric in both left and right wings) because of an increased average positron lifetime (*τ_av_*.), without changes in the peak position, in view of negligible variations in the long-lived *τ*_3_ lifetime (see Table 1). The widely expanded “tails” in the PAL spectra of under-stoichiometric nanocomposites in Figure 7a correspond to rich diversity in contribution of a long-lived Ps-related component defined by *I*_3_ intensity (the observed changes from 0.052 for g-As_40_Se_60_/PVP to 0.112 for g-As_5_Se_95_/PVP, see Table 1). In contrast, in over-stoichiometric arsenoselenides (*x* > 40), where intensities of a long-lived Ps-related component (*I*_3_) are only slightly scattered around *I*_3_ ~ 0.05, the compositionally tuned “rainbow” effects are more suppressed (see Figure 7b).

## 4. Conclusions

Volumetric effects, involving changes in atomic-deficient free-volume structure, were studied in glassy arsenoselenides g-As_x_Se_100−x_ subjected to high-energy dry and wet milling (in 0.5% PVP water solution) employing the method of positron annihilation lifetime (PAL) spectroscopy.

Preliminary analysis of milling-driven volumetric nanostructurization in g-As_x_Se_100−x_ was performed with a modified two-state simple trapping model applied under slight input from a Ps-decay channel. From this analysis, it is proved that additional positron trapping sites appear in bulky over-stoichiometric g-As_x_Se_100−x_ (x > 40) under coarse powdering, followed by a conventional pelletization procedure. In contrast, slight agglomeration of large positron traps is expected in transition from unmilled to dry-milled glassy arsenoselenides. In comparison with coarse-grained powdered and dry-milled arsenoselenides, positron trapping in wet-milled probes, representing themselves as g-As_x_Se_100−x_/PVP nanocomposites, was essentially modified by enriched contributions from Ps-decaying states stabilized in a “pure” PVP environment.

These findings were proved, assuming two inputs contributing to trapping in g-As_x_Se_100−x_ arising from distinct positron- and Ps-related sites (three-state additive simple trapping model). It is confirmed that annihilating positrons in dry-milled probes are trapped in free-volume defects stabilized intrinsically in a g-As-Se environment; however, the medium where positron annihilation occurs is cardinally changed in wet-milled probes being replaced by PVP solution.

Formalism of x3-x2-CDA (coupling decomposition algorithm) describing the conversion of bound positron–electron (positronium, Ps) states into positron traps was applied to identify free volume nanostructurization in pelletized PVP-capped g-As-Se nanocomposites, with respect to iso-compositional dry-milled ones. Under wet milling, Ps-decaying sites stabilized in inter-nanoparticulate junctions with the PVP environment replaced positron traps in dry-milled samples having defect-specific lifetimes of ~0.36–0.38 ns, the latter corresponding to multi-atomic vacancies near trigonal AsSe_3/2_ pyramids in a mostly negative environment of bridging Se atoms. Tending to under-stoichiometric Se-rich glassy arsenoselenides, g-As_x_Se_100−x_, these positron-trapping sites are agglomerated due to an abundant amount of homonuclear Se-Se bonds.

As a final accord in *positronics*, the genesis of reconstructed three-component PAL spectra with nanostructurally- and compositionally-tuned Ps-decay contribution and average positron lifetime is considered as the foundation of specific “rainbow” effects observed in a row of these spectra for pelletized PVP, and further, wet-milled, dry-milled, and unmilled g-As_x_Se_100−x_.

## Figures and Tables

**Table 1:** Fitting parameters describing unconstrained x3-term decomposed PAL spectra of pelletized unmilled, dry-milled and wet-milled g-As_x_Se_100−x_ (fitting parameters for PVP pellets [47] are included for comparison).

Samples g-As_x_Se_100−x_, x	χ = [FIT–1]	PAL Spectra Fitting Parameters					*τ_av_*.
		*τ*_1_	*τ*_2_	*τ*_3_	*I*_2_	*I*_3_	
		ns	ns	ns	a.u.	a.u.	ns
PVP pellets [47]	0.20	0.196	0.472	1.867	0.256	0.119	0.466
Unmilled, x = 5	0.04	0.217	0.377	2.000	0.300	0.011	0.285
Dry-milled, x = 5	0.04	0.210	0.369	2.137	0.340	0.011	0.285
Wet-milled, x = 5	0.03	0.212	0.495	1.764	0.303	0.112	0.472
Unmilled, x = 10	0.03	0.216	0.380	2.232	0.290	0.012	0.288
Dry-milled, x = 10	0.04	0.212	0.360	2.112	0.350	0.011	0.285
Wet-milled, x = 10	0.07	0.202	0.438	1.708	0.348	0.101	0.436
Unmilled, x = 20	0.03	0.213	0.368	2.103	0.340	0.015	0.294
Dry-milled, x = 20	0.04	0.215	0.368	2.157	0.330	0.011	0.287
Wet-milled, x = 20	0.07	0.211	0.454	1.706	0.303	0.086	0.413
Unmilled, x = 29	0.03	0.212	0.365	2.117	0.370	0.012	0.291
Dry-milled, x = 29	0.05	0.212	0.368	2.057	0.360	0.011	0.290
Wet-milled, x = 29	0.19	0.206	0.390	1.747	0.342	0.042	0.334
Unmilled, x = 40	0.01	0.193	0.358	2.091	0.620	0.007	0.309
Dry-milled, x = 40	0.05	0.202	0.371	2.087	0.540	0.015	0.322
Wet-milled, x = 40	0.07	0.212	0.428	1.891	0.400	0.052	0.386
Unmilled, x = 45	0.04	0.185	0.352	1.964	0.670	0.008	0.311
Dry-milled, x = 45	0.06	0.204	0.379	2.337	0.520	0.015	0.327
Wet-milled, x = 45	0.08	0.201	0.405	1.855	0.500	0.047	0.382
Unmilled, x = 50	0.06	0.205	0.370	2.106	0.540	0.015	0.323
Dry-milled, x = 50	0.05	0.210	0.378	2.264	0.520	0.013	0.324
Wet-milled, x = 50	0.08	0.207	0.411	1.833	0.440	0.049	0.381
Unmilled, x = 55	0.07	0.207	0.370	2.183	0.510	0.014	0.318
Dry-milled, x = 55	0.06	0.195	0.369	2.128	0.570	0.015	0.323
Wet-milled, x = 55	0.08	0.202	0.419	1.845	0.460	0.065	0.409
Unmilled, x = 60	0.05	0.194	0.359	2.108	0.580	0.015	0.318
Dry-milled, x = 60	0.05	0.204	0.382	2.339	0.520	0.015	0.329
Wet-milled, x = 60	0.06	0.216	0.442	1.903	0.400	0.059	0.406
Unmilled, x = 65	0.06	0.207	0.367	2.098	0.520	0.015	0.319
Dry-milled, x = 65	0.04	0.198	0.371	2.180	0.560	0.015	0.333
Wet-milled, x = 65	0.07	0.202	0.403	1.914	0.470	0.049	0.380

**Table 2:** Trapping modes derived from unconstrained x3-term decomposed PAL spectra of pelletized unmilled, dry-milled, and wet-milled g-As_x_Se_100−x_, assuming two-state STM with ignored Ps-decay contribution (trapping modes for PVP pellets [47] are included for comparison).

Samples g-As_x_Se_100−x_, x	Positron Trapping Modes						Ps-Decay Modes	
	*τ_av_^tr^*.	*τ_b_*	*κ_d_*	*τ*_2_ − *τ_b_*	*τ*_2_ */τ_b_*	*η_d_*	*R*_3_	*f_v_*
	ns	ns	ns^−1^	ns	a.u.	a.u.	nm	%
PVP pellets [47]	0.276	0.236	0.87	0.24	1.99	0.17	0.276	1.88
Unmilled, x = 5	0.276	0.249	0.59	0.13	1.52	0.13	0.288	0.20
Dry-milled, x = 5	0.265	0.247	0.71	0.12	1.50	0.15	0.300	0.23
Wet-milled, x = 5	0.309	0.263	0.92	0.23	1.88	0.20	0.271	1.60
Unmilled, x = 10	0.264	0.247	0.59	0.13	1.54	0.13	0.307	0.26
Dry-milled, x = 10	0.264	0.248	0.69	0.11	1.45	0.15	0.298	0.21
Wet-milled, x = 10	0.293	0.255	1.03	0.18	1.72	0.21	0.259	1.32
Unmilled, x = 20	0.267	0.249	0.68	0.12	1.48	0.15	0.297	0.29
Dry-milled, x = 20	0.266	0.250	0.65	0.12	1.47	0.14	0.301	0.22
Wet-milled, x = 20	0.292	0.257	0.84	0.20	1.77	0.18	0.259	1.13
Unmilled, x = 29	0.269	0.251	0.74	0.11	1.45	0.16	0.298	0.24
Dry-milled, x = 29	0.271	0.253	0.70	0.12	1.47	0.15	0.293	0.21
Wet-milled, x = 29	0.272	0.248	0.82	0.14	1.58	0.17	0.263	0.57
Unmilled, x = 40	0.296	0.271	1.49	0.09	1.32	0.29	0.296	0.15
Dry-milled, x = 40	0.295	0.269	1.24	0.10	1.37	0.25	0.296	0.29
Wet-milled, x = 40	0.303	0.269	1.00	0.16	1.58	0.21	0.277	0.83
Unmilled, x = 45	0.298	0.272	1.73	0.08	1.30	0.32	0.270	0.11
Dry-milled, x = 45	0.296	0.270	1.20	0.11	1.40	0.24	0.318	0.35
Wet-milled, x = 45	0.308	0.273	1.31	0.14	1.54	0.26	0.274	0.72
Unmilled, x = 50	0.295	0.271	1.19	0.10	1.36	0.24	0.298	0.29
Dry-milled, x = 50	0.299	0.274	1.12	0.10	1.38	0.23	0.311	0.30
Wet-milled, x = 50	0.301	0.268	1.10	0.14	1.53	0.23	0.272	0.74
Unmilled, x = 55	0.291	0.268	1.10	0.10	1.38	0.23	0.304	0.30
Dry-milled, x = 55	0.296	0.268	1.40	0.10	1.38	0.27	0.300	0.31
Wet-milled, x = 55	0.309	0.271	1.26	0.15	1.25	0.26	0.273	0.99
Unmilled, x = 60	0.291	0.266	1.40	0.09	1.35	0.27	0.298	0.30
Dry-milled, x = 60	0.298	0.271	1.21	0.11	1.41	0.25	0.318	0.36
Wet-milled, x = 60	0.312	0.276	1.01	0.17	1.61	0.22	0.278	0.97
Unmilled, x = 65	0.291	0.269	1.11	0.10	1.37	0.23	0.296	0.30
Dry-milled, x = 65	0.293	0.266	1.29	0.10	1.35	0.26	0.304	0.32
Wet-milled, x = 65	0.301	0.268	1.22	0.14	1.22	0.25	0.279	0.80

**Table 3:** Trapping modes derived from unconstrained x3-term decomposed PAL spectra of pelletized unmilled, dry-milled, and wet-milled g-As_x_Se_100−x_ assuming three-state additive STM.

Samples g-As_x_Se_100-x_, x	*τ_B_* ^1–2^	*κ_d_*_1_	*κ_d_*_2_	*κ_d_*_1_/*κ_d_*_2_
	ns	ns^−1^	ns^−1^	a.u.
Unmilled, x = 5	0.251	0.59	0.05	11.8
Dry-milled, x = 5	0.249	0.70	0.05	14.0
Wet-milled, x = 5	0.291	0.82	0.47	1.74
Unmilled, x = 10	0.250	0.58	0.05	11.6
Dry-milled, x = 10	0.251	0.68	0.05	13.6
Wet-milled, x = 10	0.279	0.93	0.44	2.11
Unmilled, x = 20	0.253	0.67	0.06	11.2
Dry-milled, x = 20	0.252	0.64	0.06	10.7
Wet-milled, x = 20	0.277	0.77	0.36	2.14
Unmilled, x = 29	0.254	0.73	0.05	14.6
Dry-milled, x = 29	0.256	0.70	0.05	14.0
Wet-milled, x = 29	0.257	0.78	0.18	4.33
Unmilled, x = 40	0.273	1.48	0.03	49.3
Dry-milled, x = 40	0.273	1.22	0.07	17.4
Wet-milled, x = 40	0.282	0.95	0.22	4.32
Unmilled, x = 45	0.274	1.72	0.04	43.0
Dry-milled, x = 45	0.273	1.18	0.07	16.9
Wet-milled, x = 45	0.283	1.25	0.21	5.95
Unmilled, x = 50	0.275	1.18	0.07	16.9
Dry-milled, x = 50	0.277	1.10	0.06	18.3
Wet-milled, x = 50	0.277	1.06	0.21	5.05
Unmilled, x = 55	0.271	1.09	0.06	18.2
Dry-milled, x = 55	0.272	1.38	0.07	19.7
Wet-milled, x = 55	0.287	1.18	0.29	4.07
Unmilled, x = 60	0.270	1.37	0.07	22.8
Dry-milled, x = 60	0.274	1.19	0.07	17.0
Wet-milled, x = 60	0.291	0.95	0.24	4.17
Unmilled, x = 65	0.272	1.10	0.07	15.7
Dry-milled, x = 65	0.259	1.32	0.07	18.9
Wet-milled, x = 65	0.280	1.16	0.22	5.27

**Table 4:** Matrix of expected positron annihilation channels in g-As_x_Se_100-x_-based composites.

Positron Annihilation Channel	Bulk g-As-Se (Unmilled, Unpelletized)	Cgp g-As-Se (Unmilled, Pelletized)	Fgp g-As-Se (Dry-Milled, Pelletized)	Fgp g-As-Se/PVP (Wet-Milled, Pelletized)
{DFB} state	{DFB}^g-As-Se^–int	{DFB}^g-As-Se^–int	{DFB}^g-As-Se^–int	{DFB}^g-As-Se^–int
Positron {e^+^}-traps	{e^+^}^g-As-Se^–int	{e^+^}^g-As-Se^–int	{e^+^}^g-As-Se^–int	{e^+^}^g-As-Se^–int
	-	{e^+^}^g-As-Se^–IF-1	{e^+^}^g-As-Se^–IF-2	{e^+^}^g-As-Se/PVP^–IF-3
Positronium {Ps}-traps	{Ps}^g-As-Se^–int-1	{Ps}^g-As-Se^–int-2	{Ps}^g-As-Se^–int-2	{Ps}^g-As-Se^–int-2
	-	{Ps}^g-As-Se^–FVE-1	{Ps}^g-As-Se^–FVE-2	{Ps}^g-As-Se/PVP^–FVE-3

Notes: DFB—defect-free bulky (state); int—intrinsic; GB—grain-boundary; IF—interfacial; cgp—coarse-grained powdered; fgp—fine-grained powdered; equivalent annihilation channels are yellow-colored; positron-Ps trapping-conversion channels are grey-colored.

**Table 5:** Calculated x3-x2-CDA trapping modes in dry-milled g-As_x_Se_100−x_ defined in respect to iso-compositional wet-milled g-As_x_Se_100−x_/PVP nanocomposites.

Sample	I Component		II Component		Positron Trapping Modes				
	*τ_n_*	*I_n_*	*τ_int_*	*I_int_*	*τ_av_^NP^*	*τ_b_^NP^*	*κ_d_^NP^*	*τ_int_ − τ_b_^NP^*	*τ_int_*/*τ_b_^NP^*
	ns	a.u.	ns	a.u.	ns	ns	ns^−1^	ns	a.u.
x = 5	0.210	0.573	0.357	0.310	0.261	0.245	0.69 ≅ 0.7	0.11	1.46
x = 10	0.218	0.574	0.364	0.272	0.265	0.250	0.60 ≅ 0.6	0.11	1.46
x = 20	0.216	0.561	0.357	0.201	0.264	0.249	0.63 ≅ 0.6	0.11	1.43
x = 33	0.212	0.475	0.362	0.304	0.270	0.253	0.77 ≅ 0.8	0.11	1.44
x = 40	0.194	0.334	0.352	0.472	0.287	0.263	1.35 ≅ 1.4	0.09	1.34
x = 45	0.205	0.320	0.369	0.376	0.294	0.270	1.17 ≅ 1.2	0.10	1.37
x = 50	0.211	0.351	0.368	0.403	0.295	0.274	1.08 ≅ 1.1	0.10	1.35
x = 55	0.193	0.331	0.358	0.464	0.289	0.264	1.39 ≅ 1.4	0.09	1.35
x = 60	0.201	0.359	0.367	0.418	0.291	0.266	1.21 ≅ 1.2	0.10	1.38
x = 65	0.193	0.315	0.353	0.433	0.286	0.261	1.37 ≅ 1.4	0.09	1.35

## Data Availability

The data presented in this study are available upon request from the corresponding author.
